# Synthesis of Zn(II)-Doped Magnetite Leaf-Like Nanorings for Efficient Electromagnetic Wave Absorption

**DOI:** 10.1038/srep45480

**Published:** 2017-04-03

**Authors:** Shuang Yang, Jian-Tang Jiang, Cheng-Yan Xu, Yang Wang, Yan-Yan Xu, Lei Cao, Liang Zhen

**Affiliations:** 1 School of Materials Science and Engineering, Harbin Institute of Technology, Harbin 150001, China; 2 MOE Key Laboratory of Micro-System and Micro-Structures Manufacturing, Harbin Institute of Technology, Harbin 150080, China; 3 Academy of Fundamental and Interdisciplinary Sciences, Harbin Institute of Technology, Harbin 150080, China; 4 College of Chemistry, Tianjin Normal University, Tianjin 300387, China

## Abstract

We report the thermal annealing-induced formation of ring-like structure of Zn(II)-doped magnetite from iron alkoxide leaf-like nanoplate precusor. The phase, structure and morphology of magnetite nanorings were comprehensively characterized by powder X-ray diffraction, X-ray photoelectron spectroscopy, atomic force microscope, scanning electron microscope, and transmission electron microscope. The obtained Zn(II)-doped magnetite nanorings are of 13–20 nm in edge width, 70–110 nm in short axis length and 100–150 nm in long axis length. The growth mechanism was possibly due to a combined effect of decomposition of the organic component and diffusion growth. Zn(II)-doped magnetite nanorings delivered saturation magnetization of 66.4 emu/g and coercivity of 33 Oe at room temperature. In addition, the coatings containing Zn(II)-doped magnetite nanorings as fillers exhibit excellent microwave absorption properties with a maximum reflection loss of −40.4 dB and wide effective absorbing band obtained in coating with thin thickness of 1.50 mm.

Electromagnetic wave absorbing (EMA) materials are at the core of electromagnetic interference (EMI) prevention and radar concealing (RC) technology^1^. So far, many kinds of EMA materials including ferrites, metallic powders, ceramics powders, carbon fibers, *etc*., have been studied in the past a few decades to meet the ever-increasing requirements from EMI prevention and RC technology^2^,^3^,^4^,^5^,^6^,^7^,^8^,^9^. Among these materials, magnetite is a good candidate as they present unique electromagnetic (EM) properties^6^,^7^,^8^,^9^. The electromagnetic properties of magnetite are very sensitive to their compositions, microstructures, shapes and dimensions^10^,^11^,^12^. In general, the morphology and microstructure will affect the conductivity, electric polarization and magnetic properties of magnetite, which will then influences the complex permeability and permittivity in microwave band.

Many efforts were inspired to tailor the microstructure and morphology of magnetite, aiming to optimize the permittivity, permeability and the EM matching. Specifically, a lot of attention has been focused on magnetite nanostructures in recent years^6^,^7^,^8^,^9^,^10^,^11^,^12^. Magnetite nanostructures, including nanoparticles, nanospheres, nanowires, nanorings, nanoporous structures and nanoplatelets, have been prepared by a variety of synthetic methods involving chemical co-precipitation, electrochemical, solvothermal method and iron compound decompositions, *etc*. In particular, nanorings have attracted strong interests because of their novel structure and properties^10^,^11^,^13^,^14^,^15^,^16^. Jia *et al*.^13^ first synthesized magnetite nanorings via a reduction process. The synthesized magnetite nanorings have the single-vortex magnetic state and could hold the information storage in the circulating magnetization. Chen *et al*.^14^ synthesized necklace-like magnetite nanorings by a one-step solvothermal method. Xing *et al*.^15^ reported the formation of magnetite ring-like nanostructures through oriented attachment of magnetite nanocrystal building blocks via a hydrothermal process. Fan *et al*.^16^ developed a new universal template route to fabricate Fe_3_O_4_ nanorings through a simple thermal transformation method, which have great potential in biomedicine and biomagnetic applications.

Although significant efforts have been exerted to prepare Fe_3_O_4_ nanorings, there are only a few report on the EMA properties of Fe_3_O_4_ nanorings. Recently, Tong’s group^10^ investigated the EMA properties of elliptical Fe_3_O_4_ nanorings. The coating using Fe_3_O_4_ nanorings as fillers presented maximum reflection loss (*RL*_*max*_) of −58.9 dB at 17.7 GHz, revealing the superiority of Fe_3_O_4_ nanorings for EMA applications. The ring-like structure is believed to contribute to the high dielectric/ferromagnetic loss, suggesting the feasibility to obtain enhanced EMA efficiency. However, main EMA peaks appear at high frequency band in the study, which then partially restrict the application of this catalog of materials. It is thus a challenge to obtain enhanced EM properties in Fe_3_O_4_ nanorings, which is crucial for extending the EMA application into low frequency band such as C or X band.

The magnetic and electromagnetic properties of magnetite can be tuned significantly upon doping with metal elements. Specifically, the saturation magnetization (*M*_*s*_) and the coercivity (*H*_*c*_) depend on the category and location of metal ion-dopants. Liu *et al*.^17^ successfully adjusted the *M*_*s*_ and *H*_*c*_ of Zn-doped Fe_3_O_4_ nanoparticles by controlling the Zn doping amounts. On the other hand, the substitution of Fe^2+^ by metal ions may lead to enhanced permittvity/permeability. Shimada *et al*.^18^ observed that the permeability increases with increasing Zn concentration in Ni–Zn ferrite films. On the basis of the above literatures, we could speculate that Zn ion doping can be an effective method to tailor the magnetic and EM properties of Fe_3_O_4_ nanorings.

In this work, Zn(II)-doped magnetite nanorings were prepared by thermal decomposition of the solvothermal iron alkoxide precursor. The possible formation mechanism of ring-like structrure was proposed based on time-dependent experiments. Furthermore, the magnetic and microwave absorption properties of Zn(II)-doped magnetite nanorings were evaluated. Benefiting from zinc ion-dopants and ring-like structures, the obtained magnetite nanorings exhibit excellent microwave absorption property in lower frequency.

## Results and Discussion

**Figure 1:**
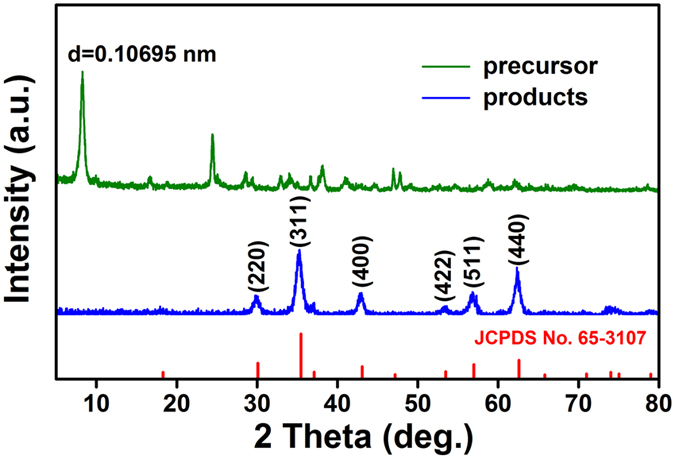
XRD patterns of iron alkoxide precursor and magnetite products. The low panel shows standard diffraction patterns of magnetite (JCPDS No. 65–3107).

The crystal structure and phase of iron alkoxide precursor and magnetite products were examined by XRD (Fig. 1). The strong XRD peak at around 10° is a typical feature from the coordination and alcoholysis of EG with the center metal ions^19^. The FTIR spectrum of the precursor is shown in Figure S1. The peak at 2500–3000 cm^−1^ could be attributed to the characteristic of the C-H stretching mode. Except the δ_H2O_ vibration at 1620 cm^−1^, the bands located below are assigned to Fe-O, C-O, C-C, and CH_2_ bonds^20^. The solvothermal precursor is thought as a kind of iron alkoxide^21^.

**Figure 2:**
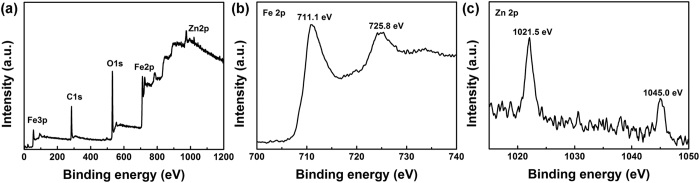
XPS spectra of the obtained magnetite from thermal annealing of iron alkoxide precursor. (**a**) Survey spectrum; (**b**) Fe 2p and (**c**) Zn 2p XPS spectra.

DSC was performed to determine the calcination temperature of iron alkoxide precusor. As shown in Figure S2, the precursor decomposed at the temperature range from 300 to 400 °C. Black products were obtained when the precursor was annealed at 350 °C for 1 h in argon atmosphere. XRD pattern of the calcined products is shown in Fig. 1. All diffraction peaks can be indexed to a face-centered cubic phase (*fcc*, space group *Fd-3m*) of magnetite (JCPDS No. 65-3107). No evident peaks of impurity phase are detected, indicating the high purity of final products. The average crystalline size of products was evaluated to be 21.1 nm based on the (311) diffraction peak using the Debeye–Scherrer formula. Fig. 2a shows XPS survey spectrum of Fe_3_O_4_ products, indicating that the products contain C, O, Fe and Zn elements. The Fe 2p XPS spectrum of Fe_3_O_4_ products is shown in Fig. 2b. The electron-binding energy of Fe 2p_3/2_ and Fe 2p_1/2_ are 711.1 and 725.8 eV, respectively, in good agreement with those previously reported^22^,^23^. As shown in Fig. 2c, there are two peaks at 1021.5 and 1045.0 eV, which are assigned to Zn 2p_3/2_ and Zn 2p_1/2_, respectively, suggesting the presence of Zn^2+^ in the Fe_3_O_4_ products^24^,^25^. Additionally, Zn content in the Fe_3_O_4_ products is determined to be about 1.37 at%. Figure S1 shows the FTIR spectrum of the black products. The peaks at 570 cm^−1^ were indicative of Fe_3_O_4_^26^, which also demonstrate that the final products are magnetite.

**Figure 3:**
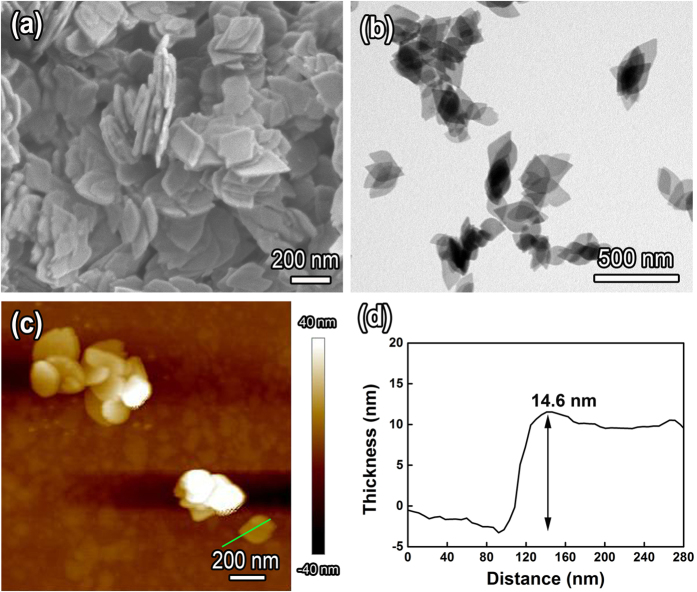
(**a**) SEM, (**b**) TEM and (**c**) AFM topography images of iron alkoxide precursor nanoplates. (**d**) Height profile of an individual nanoplate shown in Fig. 3c.

A representative SEM image in Fig. 3a shows that the precursor consists of uniform leaf-like nanoplates. These leaf-like nanoplates are of 200–300 nm in long axis length, and 110–150 nm in short axis length (Fig. 3b). The thickness of an individual nanoplate was measured by AFM and the results are shown in Fig. 3c and d. The thickness of alkoxide nanoplate is about 14.6 nm. The elemental mapping reveals that Fe, O and Zn are homogeneously distributed in each individual leaf-like nanoplate (Figure S3). The elemental mapping shows the presence of Zn in the precursor, which further demonstrates the Zn doping, in good agreement with the XPS result. It should be noted that the adding of slight amount of zinc plays a critical role in controlling the leaf-like nanoplate morphology, which has been discussed in detail in our earlier work^27^.

**Figure 4:**
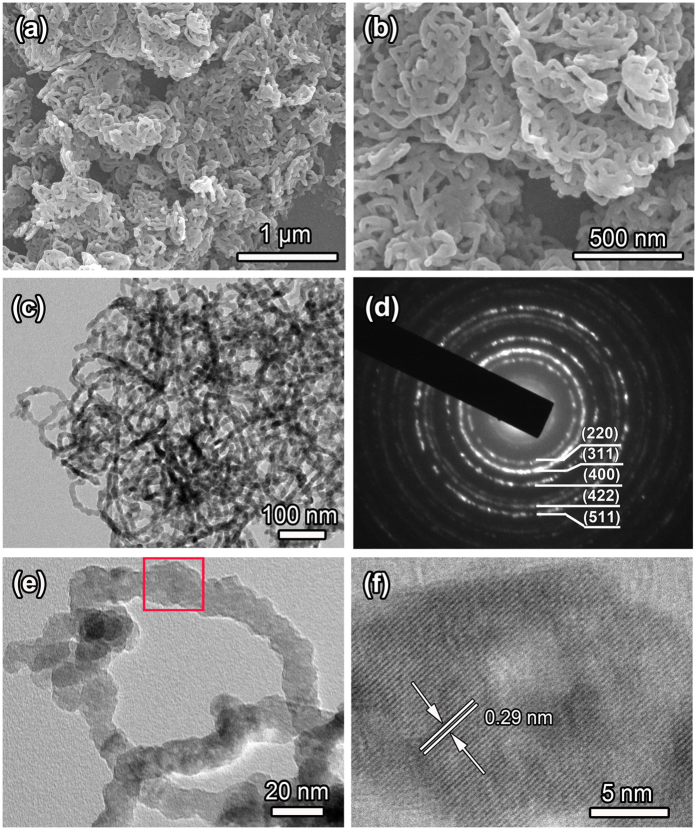
SEM and TEM characterization of leaf-like magnetite nanorings. (**a**,**b**) SEM images; (**c,e**) TEM images; (**d**) SAED pattern and (**f**) HRTEM image from boxed area in Fig. 4e.

The leaf-like nanorings shaped up after the precursors were calcined at 350 °C for 1 h in argon atmosphere, as shown in Fig. 4a and b. The obtained magnetite nanorings still kept the leaf-like configuration, and their edge width, short axis length, and long axis length were 13–20 nm, 70–110 nm, and 100–150 nm, respectively. TEM image (Fig. 4c) clearly indicates the obtained sample was leaf-like nanorings. Fig. 4e shows a high-magnification TEM image of an individual ring, demonstrating that the nanoring was composed of numerous nanoparticles. Selected-area electron diffraction (SAED) pattern of the leaf-like nanorings is presented in Fig. 4d. The concentric ring characteristic indicated the polycrystalline structure of the as-prepared magnetite nanorings. The strong rings observed in SAED pattern were indexed to the (220), (311), (400), (422), and (511) planes of the face-centered cubic magnetite, respectively. SAED results agree with that from the XRD analysis fairly well. Concurrently, the clear lattice fringes with interplanar distance of 0.29 nm were observed in the HRTEM image (Fig. 4f) of the nanorings (the red rectangle in Fig. 4e), which can be indexed as the (220) planes of the magnetite.

**Figure 5:**
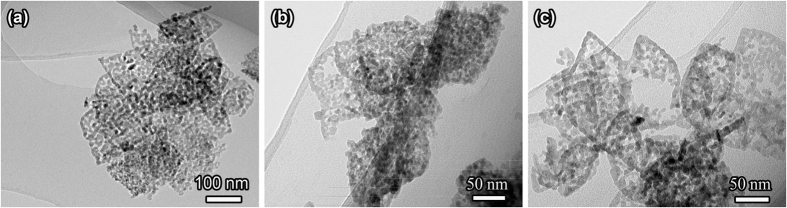
TEM images of Zn(II)-doped magnetite product obtained by calcining the precursor at 350 °C for different time in argon atmosphere: (**a**) 15 min; (**b**) 30 min; (**c**) 45 min.

To investigate the formation mechanism of Zn(II)-doped magnetite nanorings, time dependent experiments were conducted with other reaction conditions were set same as typical experiment. The products (named as intermediate) prepared with different calcining time of 15, 30, and 45 min were characterized by TEM (Fig. 5). From the TEM image shown in Fig. 5a, it is interesting that the “pores” arise in the surface of leaf-like nanoplates. As the calcining time was prolonged to 30 min (Fig. 5b), there are some small fragments due to thermal decomposition, and the “pores” of the leaf-like nanoplates surface become larger. When the calcining time was further prolonged to 45 min, the leaf-like nanorings were formed gradually, as shown in Fig. 5c. When the calcining time was further prolonged to 1 h, precursors were completely converted and leaf-like nanorings of 13–20 nm in edge width were obtained, as shown in Fig. 4. In addition, by closer observation of Figs 4e and 5a–c, it can be found that the edge width of the products increases with increasing calcination time.

**Figure 6:**
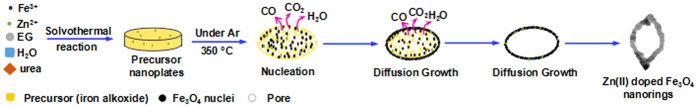
Schematic illustration of the formation of Zn(II)-doped magnetite nanorings.

Based on the above results, we propose the possible formation process of Zn(II)-doped magnetite nanorings, as schematically illustrated in Fig. 6. First, leaf-like precursor nanoplates of iron alkoxide were solvothermally synthesized using the mixture of EG and trace amount of water as solvent. The presence of trace amount of water may facilitate the decomposition of urea to create a weak alkaline condition for the formation of the iron alkoxide precursors^27^. Then, the subsequent calcining process is performed to remove the organic component and to achieve Fe_3_O_4_ phase, not tune the morphology of the products. Upon calcination in inert atmosphere, the iron alkoxide precursor decomposes to form Fe_3_O_4_ nuclei and simultaneously release a mass of gases^11^,^28^ (e.g., CO_2_, CO, and H_2_O), which would generate *in-situ* pores in the nanoplates. Thus, at the beginning of the calcining process, porous leaf-like nanoplates are obtained (Fig. 5a). The as-generated pores in thin nanoplates would induce the stress concentration. In order to decrease the stress concentration, the bigger pores have to grow up via swallowing the smaller ones^28^. Simultaneously, to minimize the interfacial energy, the as-formed Fe_3_O_4_ nuclei located in the interior of nanoplates will diffuse outwards and aggregate along the edge of the nanoplates^28^. Such changes caused the morphological evolution from nanoplates to nanorings. Eventually, Zn(II)-doped magnetite nanorings of 13–20 nm in edge width are obtained. Based on the above discussion, the morphological evolution of nanorings from nanoplates is achieved by a combined effect of decomposition of the organic component and diffusion growth of the newly formed nanocrystals.

We further investigated the effects of Zn(Ac)_2_ on the formation of Fe_3_O_4_ nanorings. As previously observed^27^, the products were a mixture of Fe_3_O_4_ nanospheres and iron alkoxide precursor nanoplates if no Zn(Ac)_2_ was added. Once few Zn(Ac)_2_ (0.04 g) was added into the reaction system, the solvothermal precursors were nanoplates with 110–200 nm in long axis, 50–150 nm in short axis (Figure S4a). As the amount of Zn(Ac)_2_ was increased to 0.24 g, nanoplates with 210–340 nm in long axis and 110–210 nm in short axis were produced, as shown in Figure S4b. XRD patterns of all products were no detectable difference when varied amounts of Zn(Ac)_2_ were added, regardless the difference in morphology (Figure S5). TG/DSC results (Figure S6) revealed that the precursors prepared via process using different amounts of Zn(Ac)_2_ decomposed and converted completely when annealed at 350 °C, but the products present different morphologies. The products prepared with 0.04 g of Zn(Ac)_2_ translated to nerve-like nanowires (Figure S7a) after calcined at 350 °C for 1 h in argon atmosphere (named as Fe_3_O_4_ nanowires (Zn_0.04_)). While the precursors prepared with 0.24 g of Zn(Ac)_2_ translates to nanorings, as shown in Figure S7b. SEM observation suggested that these rings are 25–40 nm in edge width, 75–150 nm in short axis and 120–300 nm in long axis (named as Fe_3_O_4_ nanorings (Zn_0.24_)). All products are Fe_3_O_4_ as XRD analysis indicates (Figure S8), regardless the difference in morphology. The XPS analysis shows that Zn content in the products prepared with 0.04 and 0.24 g of Zn(Ac)_2_ was 0.48 at% and 3.45 at%, respectively (Figure S9). The above results clearly suggest that the introduction of moderate Zn^2+^ is indispensable for the formation of Fe_3_O_4_ nanorings.

**Figure 7:**
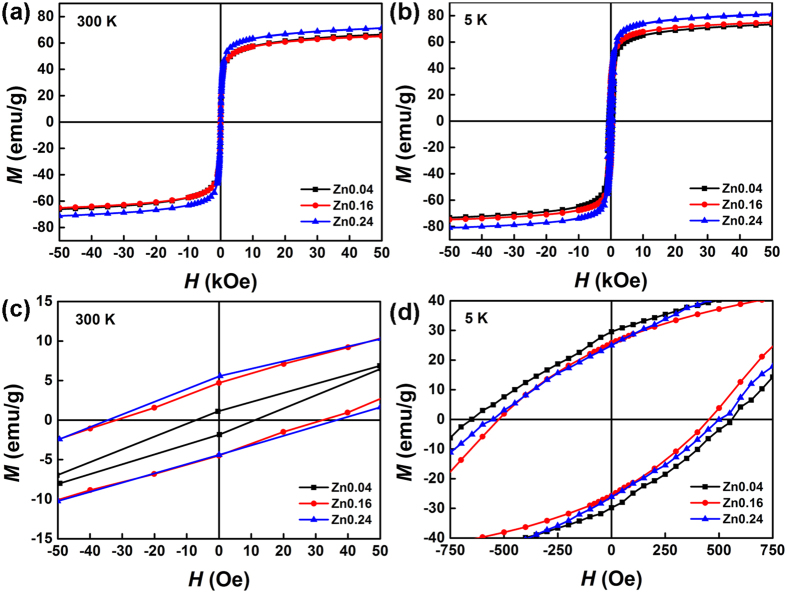
(**a**,**b**) Magnetic hysteresis loops of products prepared with different amounts of Zn(Ac)_2_ at 300 and 5 K; (**c**,**d**) Magnified hysteresis loops near zero field.

The magnetic properties of products prepared with different amounts of Zn(Ac)_2_ were measured at 5 and 300 K (Fig. 7). The saturation magnetization (*M*_*s*_) of the obtained magnetite increased with the increasing Zn concentration, while Zn_0.16_ (66.4 emu/g) and Zn_0.04_ (66.0 emu/g) have lower *M*_*s*_ than that of Zn_0.24_ (71.3 emu/g) at 300 K (Fig. 7a). As shown in Fig. 7b, *M*_*s*_ for Zn_0.04_ samples was 73.3 emu/g at 5 K and slightly increasing *M*_*s*_ was observed at Zn_0.16_ (74.8 emu/g). As for Zn_0.24_ with highest Zn content, *M*_*s*_ reached at 81.2 emu/g at 5 K. The *H*_*c*_ of Zn_0.04_, Zn_0.16_ and Zn_0.24_ samples were 12,33 and 36 Oe at 300 K, respectively (Fig. 7c), much lower than that of bulk magnetite (115–150 Oe). The lower *H*_*c*_ of the Zn(II)-doped magnetite may be attributed to small particle size of magnetite. The fine crystalline size (specifically, ~21.1 nm for Zn_0.16_) could cause spin frustration on the surface, resulting in ferromagnetic behavior^29^. The *H*_*c*_ of Zn_0.04_, Zn_0.16_ and Zn_0.24_ samples were 562, 436 and 493 Oe at 5 K, respectively (Fig. 7d). It can be found that the *M*_*s*_ and *H*_*c*_ increases when the temperature decreases. As reported Liu *et al*., it attributed this to thermal fluctuations as well as the hysteresis feature disappears^30^.

The magnetic transition was investigated by measuring zero-field cooled curves (ZFC) and field cooled (FC), and the results are shown in Figure S10. For Zn_0.04_ and Zn_0.16_ samples, the ZFC and FC curves don’t intersect at all. So the blocking temperature (*T*_*B*_) of Zn_0.04_ and Zn_0.16_ samples are above the room temperature, unlike those for Zn_0.24_ samples (*T*_B_ ≈280 K) can be deduced (Figure S10). As the previously reports^31^, no intersections between ZFC and FC curves could occur because only a slight amount of zinc was doped into the magnetite.

**Figure 8:**
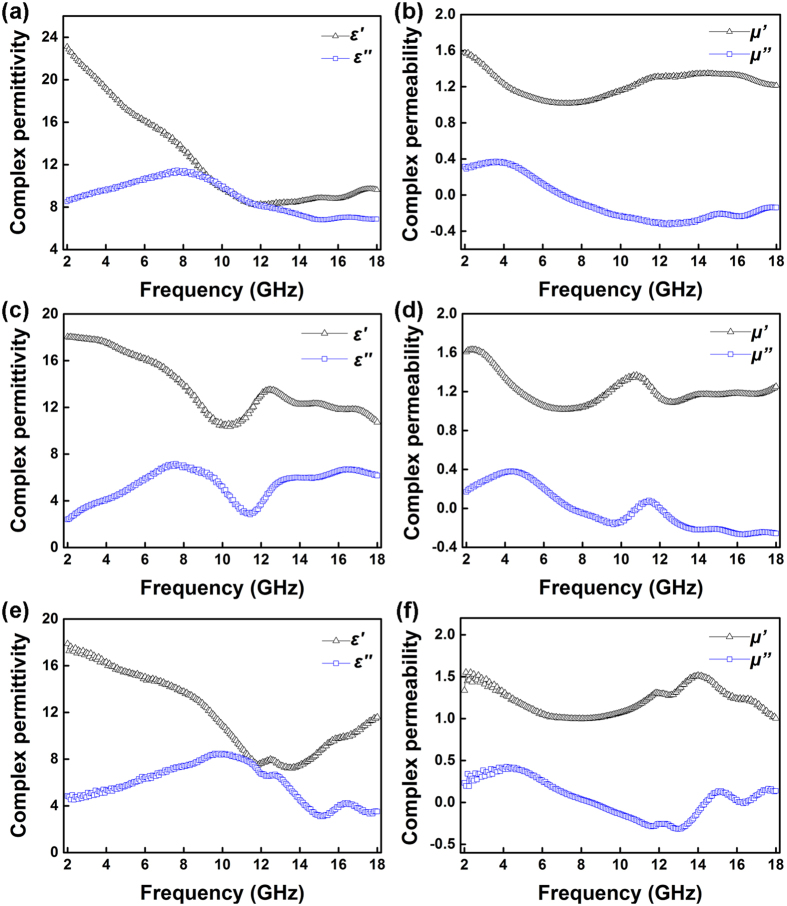
Frequency dependence of complex permittivity and complex permeability in 2–18 GHz range of wax-matrixed specimens containing 75 wt% products (magnetite nanowires or nanorings) prepared with different amounts of Zn(Ac)_2_: (**a**,**b**) 0.04 g; (**c**,**d**) 0.16 g; (**e**,**f**) 0.24 g.

The electromagnetic properties of composite specimen containing products prepared with different amounts of Zn(Ac)_2_ as fillers were also measured on a vector network analyzer (VNA) in 2–18 GHz. Fig. 8 shows the frequency dependence of the complex permittivity and complex permeability of the specimen. As seen from Fig. 8a,c and e, a dielectric relaxation is observed at the range of 6–12 GHz, as a descending of *ε′* together with a peak of *ε″* is observed. Compared with the previous research^1^,^8^,^32^,^33^, the dielectric relaxation is much more intense in the current study, which is believed to be originated from to enhanced charge polarization. The number of defective sites that serve as polarized centers increases significantly after zinc ions adoption, which thus enhances the space charge polarization^34^. Moreover, the presence of numerous nanoparticles (Fig. 4e) brings up large interface area, which will induce enhanced over-all interface polarization^35^,^36^. Hence, the substitution of Fe^2+^ by Zn^2+^ leads to enhanced polarization which then contributes to increased permittivity. On the other hand, plenty of nanorings build a conductive network for electrons hopping and migrating^37^, which could lead to increased conductivity and thus contributed enhanced electric polarization^38^. As previously reported by Tong *et al*.^10^ ring-like configuration of Fe_3_O_4_ exhibit four effective paths that corresponding to four Debye relaxation processes, as confirmed by Cole–Cole plots. A segment contains four superimposed semicircles similar to that in ref.^10^ was also observed in the present study (Figure S11), revealing the effect of conducting networks. Apart from the relaxation, a resonance was observed at about 11.5–12.5 GHz as shown in Fig. 8c and e, but not in Fig. 8a. Specifically, the resonance observed in Fig. 8c was more intense than that in Fe_3_O_4_ nanorings (Zn_0.16_). The resonance is believed to be related to the nanorings morphology of Zn(II)-doped magnetite. When used as fillers, the tubular distribution and the leaf-like morphology of Fe_3_O_4_ nanorings would tend to induce current when exposed to electromagnetic wave, leading to dielectric resonance in the curve of permittivity^38^. The diversity in resonance intensity may be related to the difference in nanorings’ size. As discussed above, zinc ion-dopants and ring-like structure could facilitate multiple dielectric losses.

Fig. 8b,d and f illustrate the frequency dependence of the complex permeability of the composites. The *μ*’ decreases apparently in the 2–6 GHz band, while *μ*”increases in 2–4 GHz band and reaches at peak at around 4 GHz, as shown in Fig. 8b,d or f, indicating the occurrence of ferromagnetic resonance in the band. According to the previous research^39^,^40^, this resonance emerges at 2–6 GHz band is originates from natural resonance. However, it exhibits much higher resonances frequency (*f*_*r*_) to compare with that of the magnetite nanoparticles^39^. The shift of *f*_*r*_ towards higher frequency is attributed to either the small size effect or high shape anisotropy^40^. Negative *μ*” can be seen when frequency is higher than 8 GHz as shown in Fig. 8b,d and f, which is attributed to left-hand effect related to the asymmetrical ring like structure^41^,^42^. Additionally, a resonance is observed at about 11.5 or 12.5 GHz, as shown in Fig. 8d or f. The resonance intensity is also found related to the size of Fe_3_O_4_ nanorings. As previously reported^10^, Fe_3_O_4_ nanorings could suppress the eddy current effect for the presence of the center hole. On the other hand, Fe_3_O_4_ nanorings, acting as plasmonic structures, induce plasmon resonance and enhance permeability^10^. Hence, Fe_3_O_4_ nanorings generate strong resonance at about 11.5 or 12.5 GHz, which was also attributed to the leaf-like nanorings structure of fillers.

**Figure 9:**
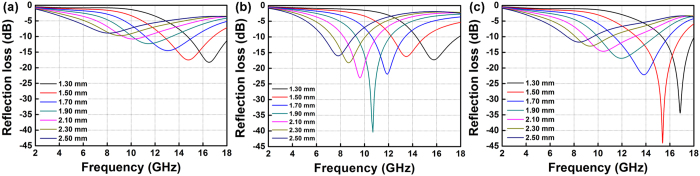
Reflection loss curves for the products prepared with different amounts of Zn(Ac)_2_: (**a**) 0.04 g; (**b**) 0.16 g; (**c**) 0.24 g.

To reveal the EMA properties of magnetite nanowires or nanorings, the reflection loss (*RL*) of corresponding coatings of various thicknesses was calculated using the measured EM parameters according to the transmit line theory as shown in Fig. 9. Coatings using Fe_3_O_4_ nanowires (Zn_0.04_) as filler present *RL*_*max*_ of −18.3 dB at 16.5 GHz (Fig. 9a). As shown in Fig. 9b, coatings containing Fe_3_O_4_ nanorings (Zn_0.16_) as fillers exhibit optimized EMA performances, as *RL*_*max*_ of −40.4 dB is observed at 10.7 GHz when a thin thickness of 1.9 mm is applied. Concurrently, the effective absorbing band (EAB, *RL*<−5 dB) covers the 8–14 GHz band. When slightly increases the thickness to 2.5 mm, an EAB (*RL*<−5 dB) covering 4.2–10.8 GHz band is obtained, suggesting excellent EMA performances in C band. When Fe_3_O_4_ nanorings (Zn_0.24_) was used as fillers, *RL*_*max*_ shifts to higher frequency to compare with the case of Zn_0.16_, as a *RL*_*max*_ of −43.9 dB at 15.4 GHz was obtained in a very thin coating (1.5 mm). (Fig. 9c). As calculation suggested, Fe_3_O_4_ nanorings (Zn_0.16_) are found excellent candidates for EMA fillers used in the highly-concerned C-X band since high *RL*, thin thickness (around 2 mm) and wide EAB were simultaneously achieved.

The location of *RL*_*max*_ is related to the electromagnetic properties of the coating at set thickness, according to the quarter-wavelength cancellation^43^:


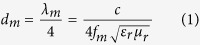


where *d*_*m*_ is the matching thickness of the coating (equals to 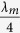), *λ*_*m*_ is the wavelength of microwave, and *c* is the light speed. According to the above formula (equation (1))^43^, large permittivity and permeability is benefit to compel *RL* peaks’ shifting to lower frequency. The magnetite nanorings that used as EMA fillers in the current study present high permittivity and permeability and thus possess the potential to present excellent EMA efficiency at low frequency when set thickness is applied. Higher *RL*_*max*_, lower *f*_*m*_ as well as wider EAB was obtained when Fe_3_O_4_ nanorings (Zn_0.16_) were used as filler, to compare with other coating as shown in Table S1. The zinc ion-dopants and ring-like structures alter the electric polarization and ferromagnetic resonances and thus leads to enhanced permittivity and permeability to compare with other structures of magnetite.

In summary, we proposed a facile synthetic strategy to prepare Zn(II)-doped magnetite nanorings through thermal annealing of iron alkoxide leaf-like nanoplates, and their application as EMA fillers was evaluated. The formation mechanism of Zn(II)-doped magnetite nanorings was possibly due to a combined effect of decomposition of the organic component and diffusion growth. The saturation magnetization of Zn(II)-doped magnetite nanorings was 66.4 emu/g at room temperature. The doping a slight amount of zinc had little impact on the magnetic properties of magnetite nanorings. Furthermore, the coating using the prepared magnetite nanorings as fillers present excellent microwave absorption property. A *RL*_*max*_ of −40.4 dB and wide EAB (8~14 GHz) was achieved in a thin coating (1.5 mm), indicating the superiority of this catalogy of materials used as EMA fillers in C-X band. The excellent EMA performance is attributed to the zinc ion-dopants and ring-like structures, which generate the space charge polarization, electric polarization and dielectric/ferromagnetic loss. The study reveals that Zn(II) doping as well as the ring-like structures present new route for excavating the potential of magnetite as EMA fillers.

## Methods

### Synthesis of Zn(II)-doped magnetite nanorings

All the reagents were of analytical grade and were used as received without further purification. First, iron alkoxide precursor nanoplates with leaf-like morphology were prepared by a solvothermal process. 28.0 mL of ethylene glycol (EG) and 1.0 mL of distilled water were used for the mixture solvent. In a typical synthesis, 0.290 g of iron chloride hexahydrate (FeCl_3_·6H_2_O), 0.16 g of zinc acetate dihydrate (Zn(Ac)_2_·2H_2_O) and 0.600 g of urea ((NH_2_)_2_CO) were dissolved into the above-mentioned mixed solution. Then, the solution was sealed into a 40 mL Teflon-lined autoclave, heated at 180 °C for 6 h. The yellow precursors were collected by high speed centrifugation, washed with ethanol and distilled water for several times, and dried at 60 °C in air. The precursors were calcined at 350 °C for 1 h in argon atmosphere to obtain magnetite (named as Fe_3_O_4_ nanorings (Zn_0.16_)).

### Characterization

X-ray diffraction (XRD) analyses were recorded on a Rigaku D/max 2500 diffractometer with Cu *K*_*α*_ radiation (*λ* = 0.15418 nm). Fourier transform infrared (FTIR) pattern was measured using a Nicolet 60-SXB FT-IR spectrometer. The morphologies and microstructures of samples were examined with a field-emission scanning electron microscope (FE-SEM, FEI Quanta 200 F), and a transmission electron microscope (TEM, JEOL JEM 2100). Thermogravimetry and differential thermal analysis (TG-DSC) was performed in a temperature ramping rate of 10 °C min^−1^ from 25 to 850 °C in N_2_ atmosphere (SDT Q600 V20.9 Build 20). X-ray photoelectron spectra (XPS) were performed using a K-Alpha spectrometer (Thermofisher Scienticfic Company). A drop of ethanol containing the magnetite nanorings was placed on a Si substrate, and then naturally dried in air prior to characterization by atomic force microscopy (AFM, Bruker Dimension ICON-PT).

### Static magnetic and electromagnetic measurements

Magnetic measurements were carried out on Quantum Design physical properties measurement system (PPMS) at 5 and 300 K with the field sweeping from −50 to 50 kOe. Zero-field cooled (ZFC) and field-cooled (FC) magnetization curves were recorded between 5 and 300 K in an applied magnetic field of 100 Oe. Zn(II)-doped magnetite/paraffin composite samples were prepared by uniformly mixing the nanorings in a paraffin matrix and then pressing the mixture into a cylindrical shaped compact. The fabricated cylindrical shaped compact is coaxial toroidals with outer diameter of 7 mm, inner diameter of 3 mm, and thicknesses of 3–3.5 mm. The electromagnetic parameters of the microstructures or nanostructures composite samples with 75 wt% of the Zn(II)-doped magnetite were measured in 2–18 GHz range by using an Agilent N5230A vector network analyzer.

## Additional Information

**How to cite this article**: Yang, S. *et al*. Synthesis of Zn(II)-Doped Magnetite Leaf-Like Nanorings for Efficient Electromagnetic Wave Absorption. *Sci. Rep.* **7**, 45480; doi: 10.1038/srep45480 (2017).

**Publisher's note:** Springer Nature remains neutral with regard to jurisdictional claims in published maps and institutional affiliations.

## Supplementary Material

Supplementary Information
