# Supplementary material for: Synthesis of Zn(II)-Doped Magnetite Leaf-Like Nanorings for Efficient Electromagnetic Wave Absorption
Source: Sci Rep. 2017 Apr 3;7:45480. doi: 10.1038/srep45480 (PMC5377309; doi:10.1038/srep45480)
Supplement: Supplementary Information [file srep45480-s1.doc]

*Supplementary Information for*

Synthesis of Zn(II)-Doped Magnetite Leaf-Like Nanorings for Efficient Electromagnetic Wave Absorption

Shuang Yang^1,2^, Jian-Tang Jiang^1,2^, Cheng-Yan Xu^1,2^, Yang Wang^3^, Yan-Yan Xu^4^, Lei Cao^1,2^ & Liang Zhen^1,2^

^1^School of Materials Science and Engineering, Harbin Institute of Technology, Harbin 150001, China. ^2^MOE Key Laboratory of Micro-System and Micro-Structures Manufacturing, Harbin Institute of Technology, Harbin 150080, China. ^3^Academy of Fundamental and Interdisciplinary Sciences, Harbin Institute of Technology, Harbin 150080, China. ^4^College of Chemistry, Tianjin Normal University, Tianjin 300387, China.

Correspondence and requests for materials should be addressed to L.Z. ([lzhen@hit.edu.cn](mailto:lzhen@hit.edu.cn)) or J.-T.J. (jjtcy@hit.edu.cn)


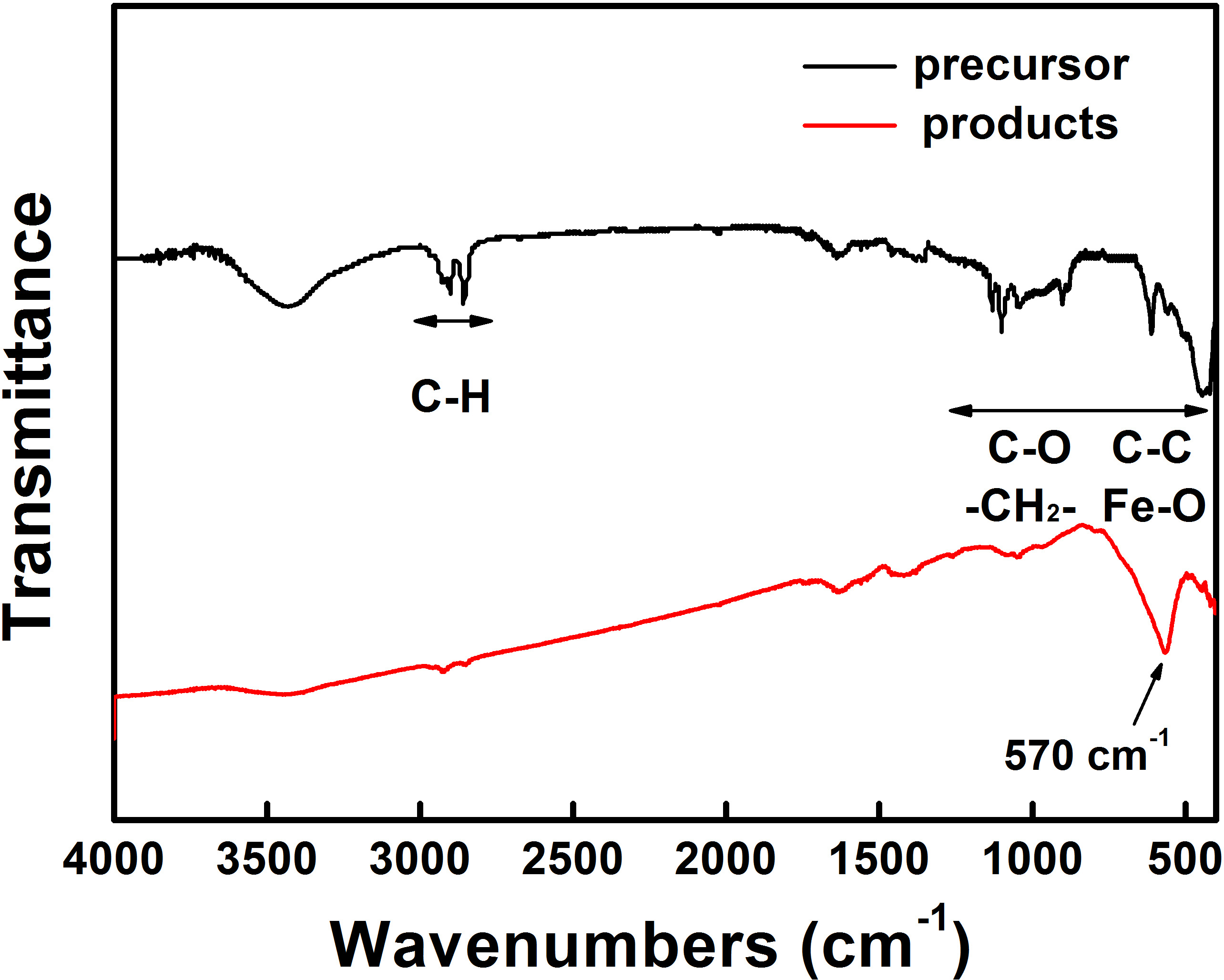


**Figure S1.** FTIR spectra of iron alkoxide precursor and magnetite product.


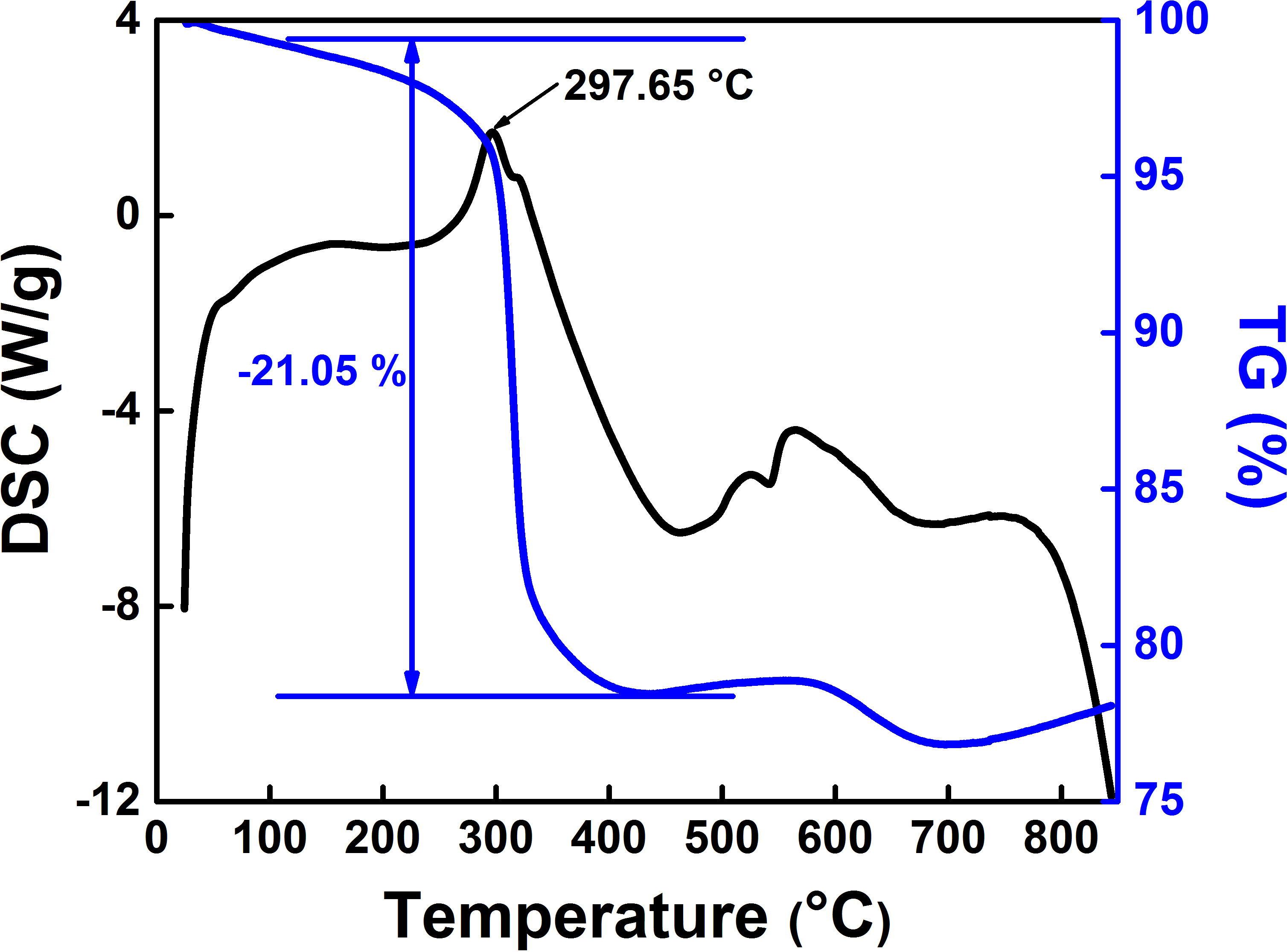


**Figure S2.** TG and DSC curves of iron alkoxide precursor.


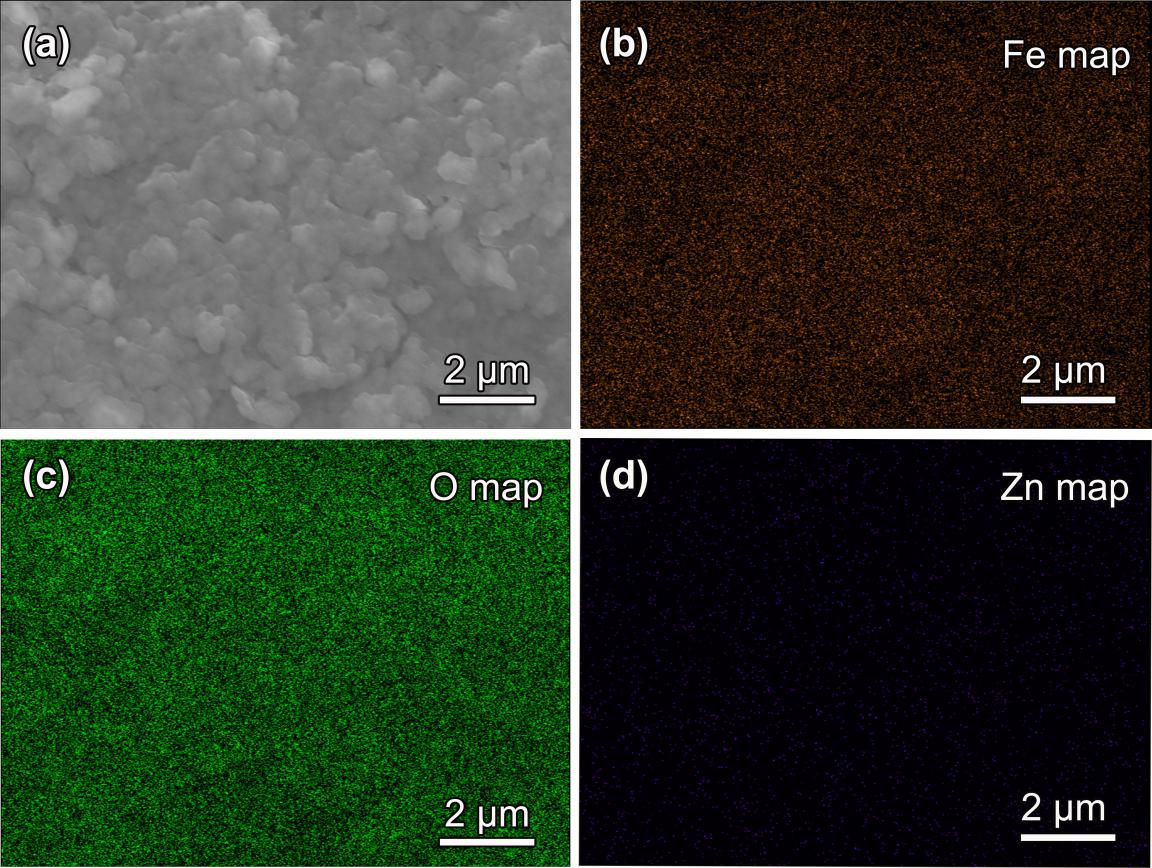


**Figure S3.** (a) SEM image of iron alkoxide precursor; (b-d) Element mapping images of Fe (b), O (c) and Zn (d).


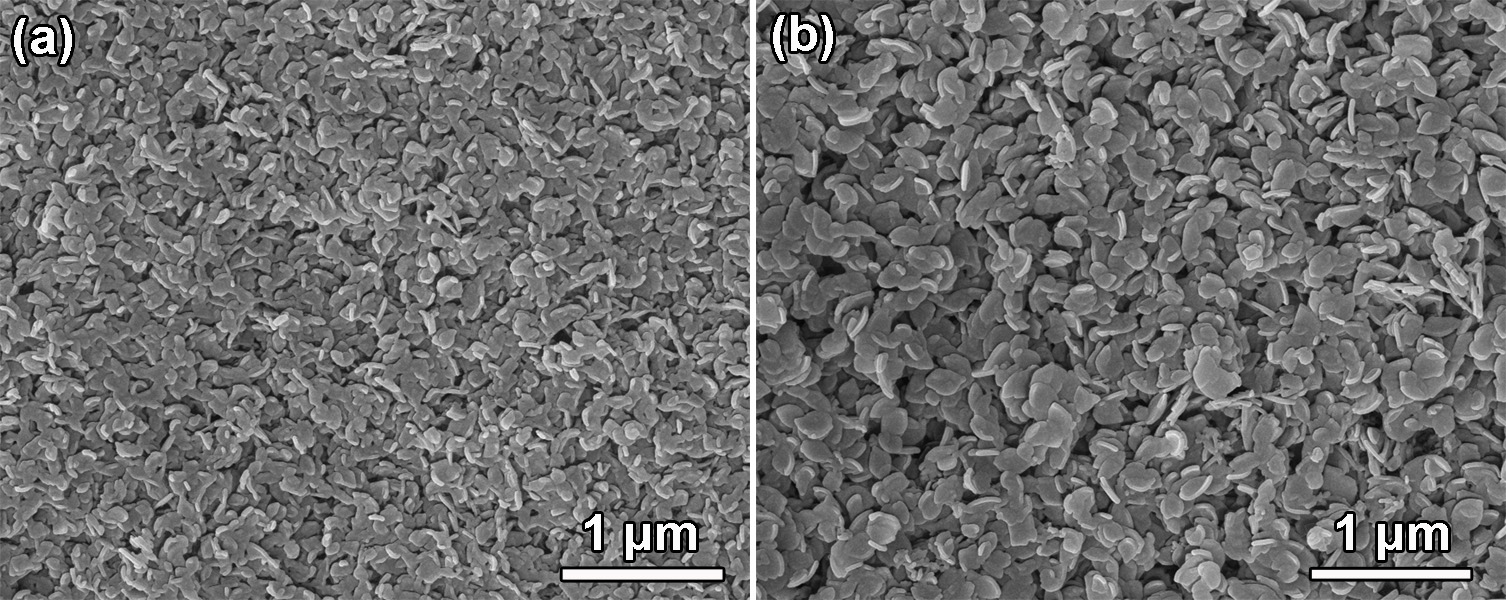


**Figure S4.** SEM images of iron alkoxide precursor nanoplates prepared with different amounts of Zn(Ac)_2_: (a) 0.04 g; (b) 0.24 g.


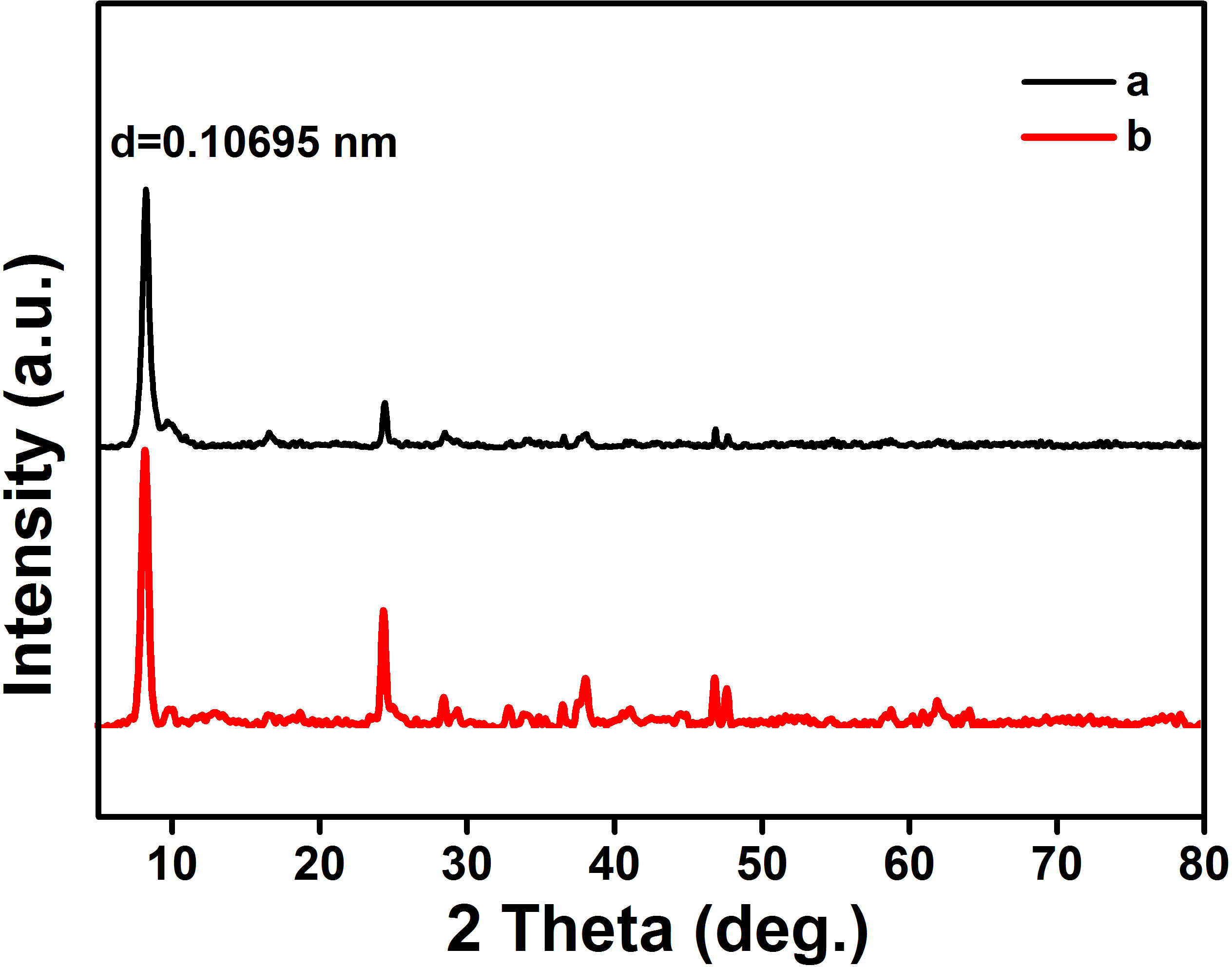


**Figure S5.** XRD patterns of iron alkoxide precursor nanoplates prepared with different amounts of Zn(Ac)_2_: (a) 0.04 g; (b) 0.24 g.


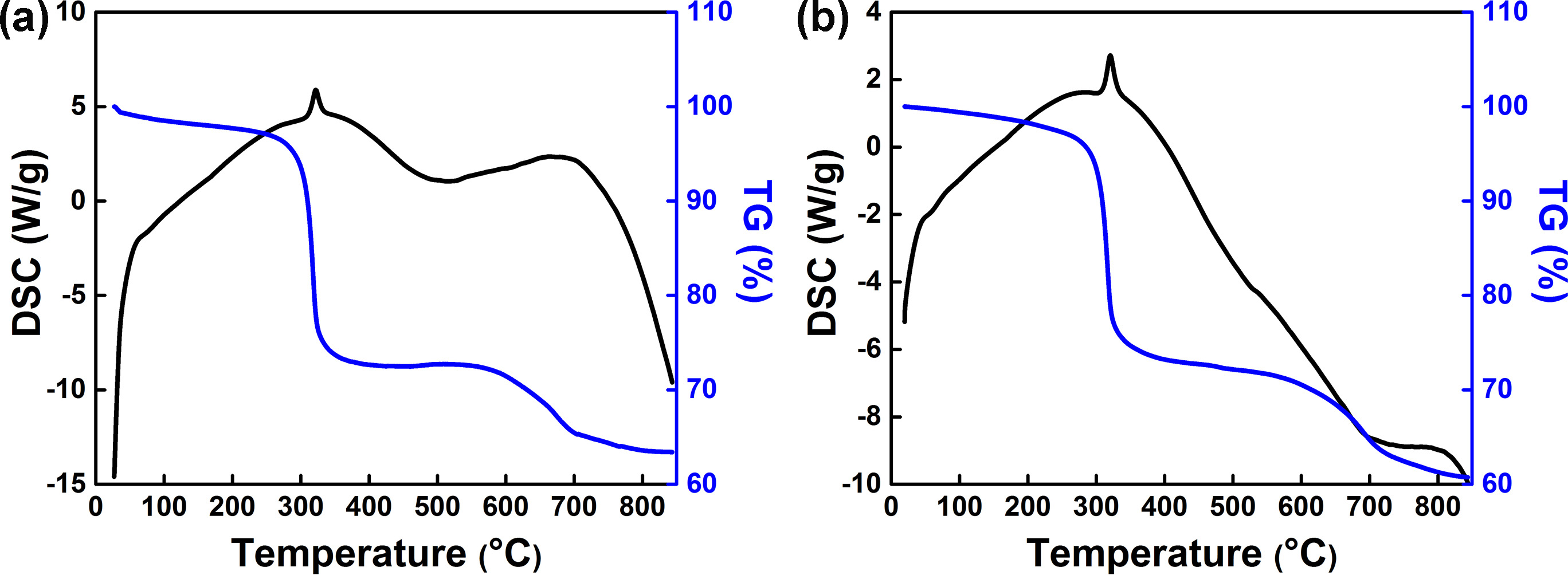


**Figure S6.** TG and DSC curves of iron alkoxide precursor prepared with different amounts of Zn(Ac)_2_: (a) 0.04 g; (b) 0.24 g.


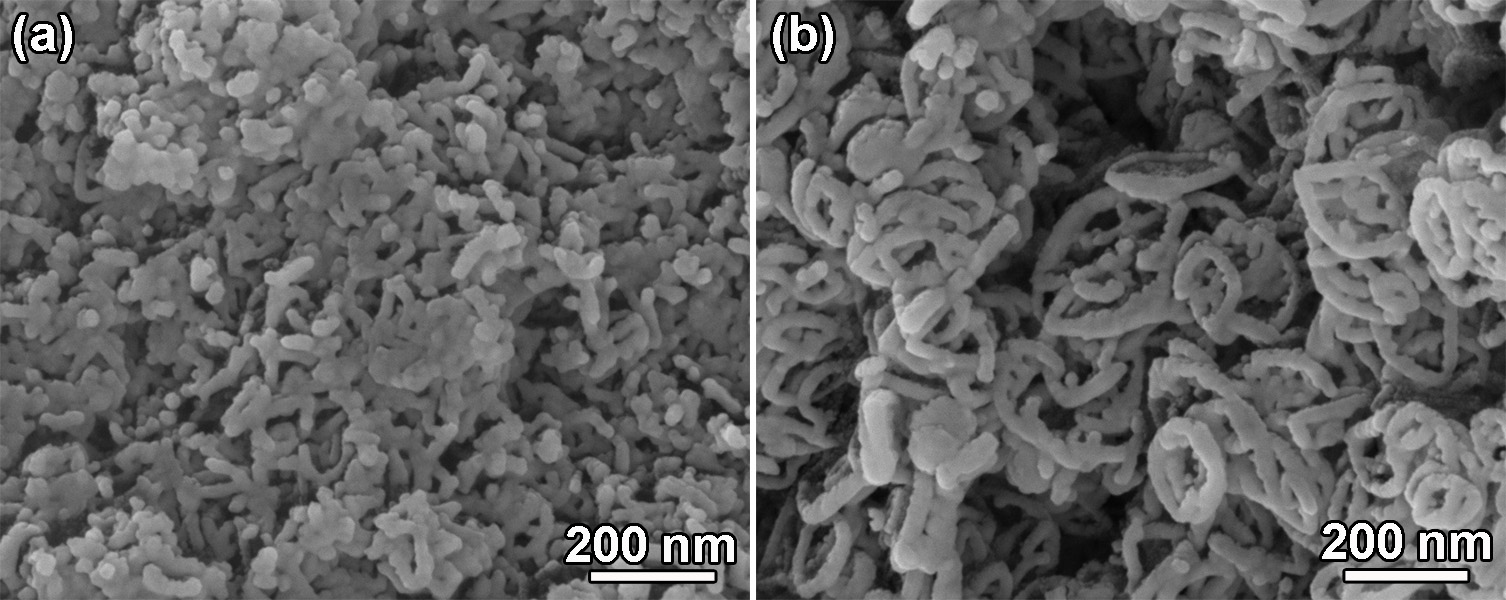


**Figure S7.** SEM images of magnetite prepared with different amounts of Zn(Ac)_2_: (a) 0.04 g; (b) 0.24 g.


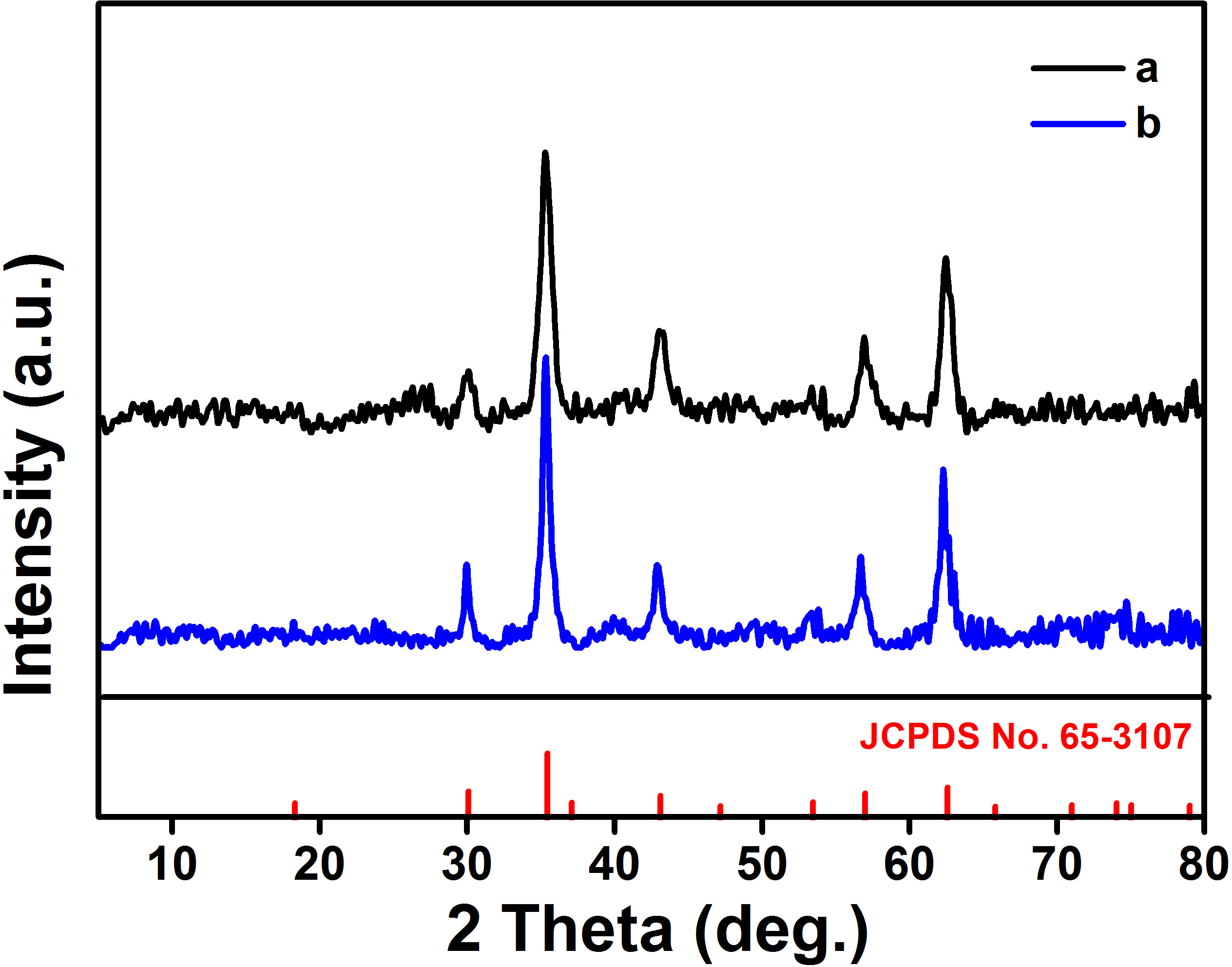


**Figure S8.** XRD patterns of magnetite prepared with different amounts of Zn(Ac)_2_: (a) 0.04 g; (b) 0.24 g. The low panel shows standard diffraction patterns of magnetite (JCPDS No. 65-3107).


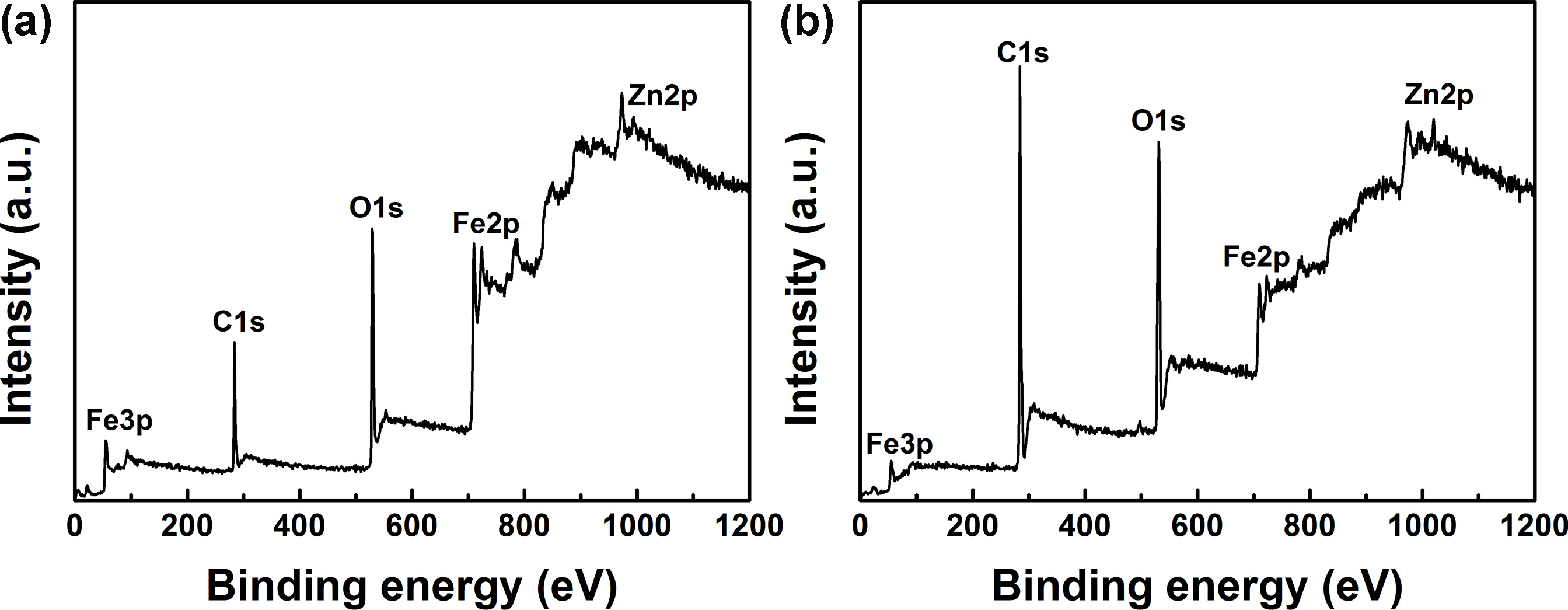


**Figure S9.** XPS survey spectra of the obtained magnetite prepared with different amounts of Zn(Ac)_2_: (a) 0.04 g; (b) 0.24 g.


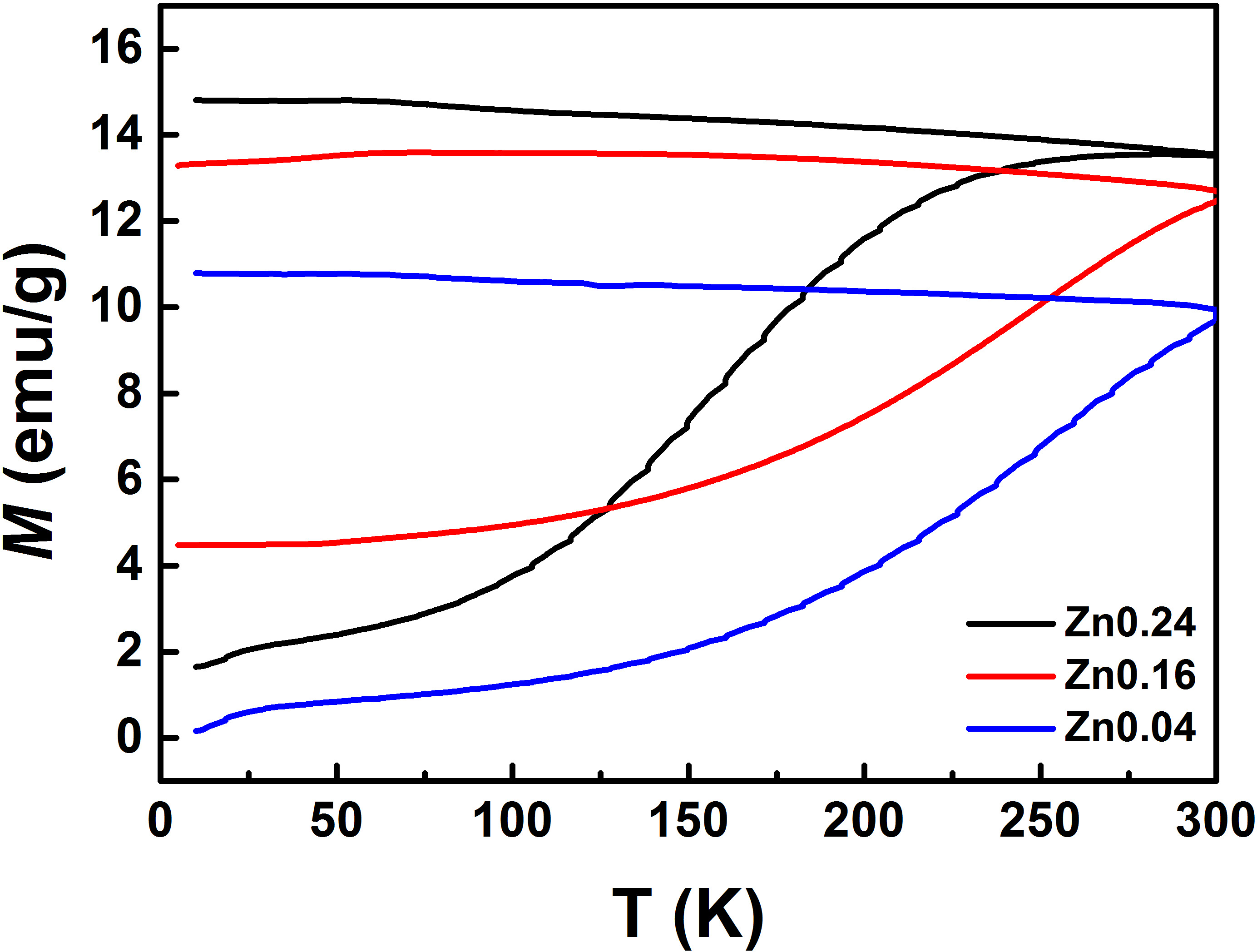


**Figure S10.** Zero-field-cooled (ZFC) and field-cooled (FC) curves of products prepared with different amount of Zn(Ac)_2_.

**
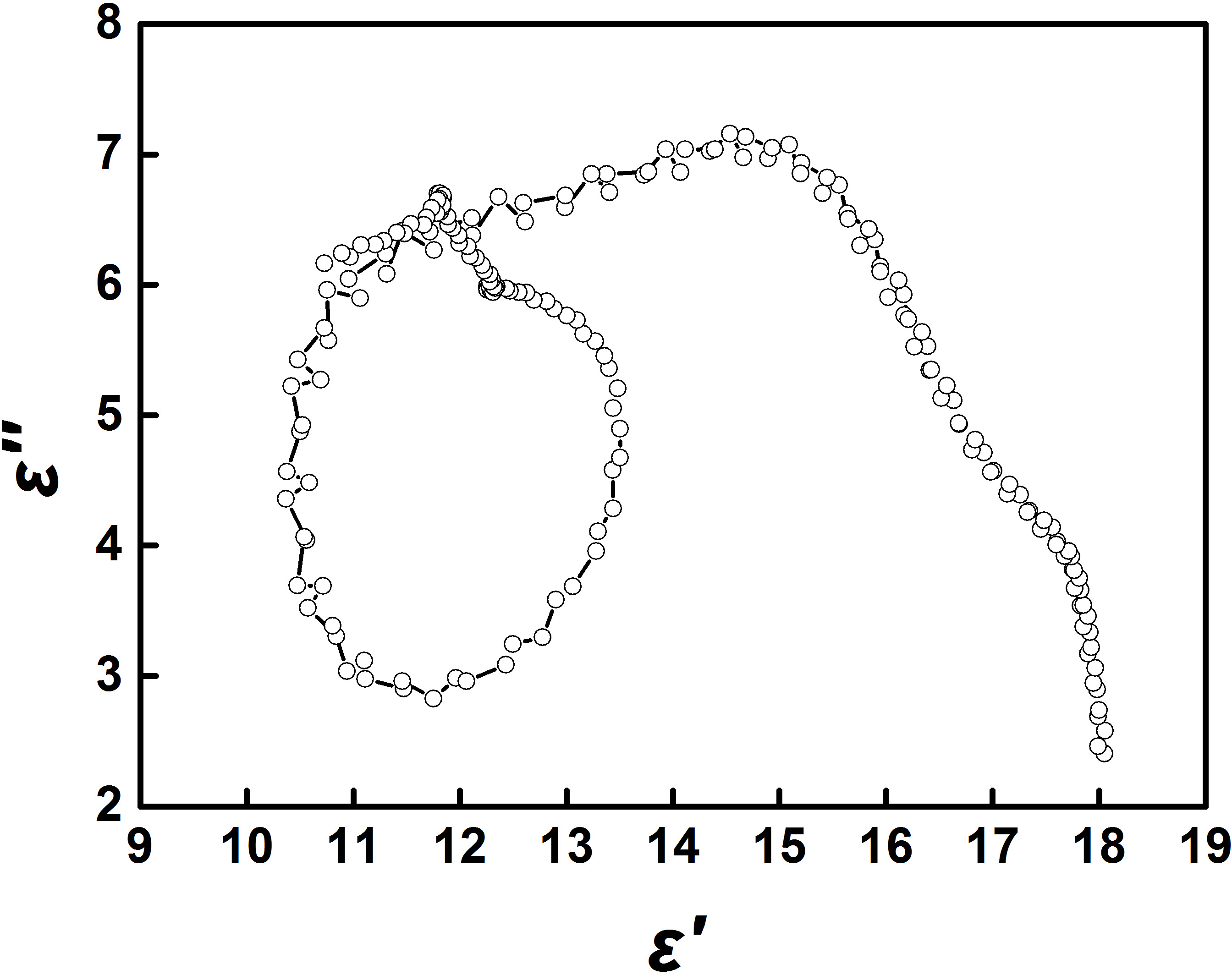
**

**Figure S11.** Cole–Cole semicircles (*ε′* versus *ε″*) for magnetite prepared with 0.16 g of Zn(Ac)_2_.

**Table S1.** Comparison of the microwave absorption properties of Fe_3_O_4_ materials reported in other studies.

| Samples | *V* (%) | *RL_max_*  (dB) | *f_m_* (GHz) | *d_m_* (mm)  (*R_L_*< –20 dB) | Frequency range (GHz)  (*R_L_*< –20 dB) | refs. |
| --- | --- | --- | --- | --- | --- | --- |
| Fe_3_O_4_ octahedral nanocrystallines | 40 | –47 | 3.1 | 3.5–6.8 | 2.0–4.3 | 1 |
| Fe_3_O_4_ dendritic microstructures | 28 | –53.0 | 2.2 | 3.0–5.0 | 2.2–4.0 | 2 |
| Fe_3_O_4_ circular  nanorings | 20 | –20 | 13.0 | / | / | 3 |
| Fe_3_O_4_ nanorings | 17 | –29.0 | 15.1 | 2.0–2.5 | 11.5–15.7 | 4 |
| Fe_3_O_4_ nanorings | 11 | –50.1 | 7.84 | 2.0–10.0 | 2.36–16.4 | 5 |
| Fe_3_O_4_ nanorings  (Zn_0.24_) | 32 | –43.9 | 15.4 | 1.3–1.7 | 13.3–17.2 | this work |
| Fe_3_O_4_ nanorings  (Zn_0.16_) | 32 | –40.4 | 10.7 | 2.1–2.5 | 9.3–12.2 | this work |

*V*: volume fraction of absorber; *RL_max_*: maximum *RL*; *f_m_*: frequency where *RL_max_* observed; *d_m_*: the coating thickness.

**References**

1. Kong, J. *et al*. Electromagnetic wave absorption properties of Fe_3_O_4_ octahedral nanocrystallines in gigahertz range. *Appl. Phys. A* **105,** 351-354 (2011).
2. Sun, G. B., Dong, B. X., Cao, M. H., Wei, B. Q. & Hu, C. W. Hierarchical dendrite-like magnetic materials of Fe_3_O_4_, γ-Fe_2_O_3_, and Fe with high performance of microwave absorption. *Chem. Mater.* **23,** 1587-1593 (2011).
3. Yong, Y., Yang, Y., Wen, X. & Jun, D. Microwave electromagnetic and absorption properties of magnetite hollow nanostructures. *J. Appl. Phys.* **115,** 17A521 (2014).
4. Wu, T. *et al*. Facile hydrothermal synthesis of Fe_3_O_4_/C core−shell nanorings for efficient low-frequency microwave absorption. *ACS Appl. Mater. Interfaces* **8,** 7370-7380 (2016).
5. Tong, G. *et al*. Tunable dielectric properties and excellent microwave absorbing properties of elliptical Fe_3_O_4_ nanorings. *Appl. Phys. Lett.* **108,** 072905 (2016).
